# Comparison of ^111^In Leakage from Labeled Endocardial and Epicardial Cells: Impact on Modeling Viability of Cells to Be Transplanted into Myocardium

**DOI:** 10.1155/2011/472375

**Published:** 2011-04-26

**Authors:** Kimberley J. Blackwood, Jane Sykes, Lela Deans, Gerald Wisenberg, Frank S. Prato

**Affiliations:** ^1^Imaging Program, Lawson Health Research Institute, 268 Grosvenor Street, London, ON, Canada N6A 4V2; ^2^Department of Medical Biophysics, University of Western Ontario, London, ON, Canada N6A 3K7; ^3^Division of Cardiology, London Health Sciences Centre, London, ON, Canada N6A 5W9

## Abstract

*Introduction*. Previously we proposed a cellular imaging technique to determine the surviving fraction of transplanted cells in vivo. Epicardial kinetics using Indium-111 determined the Debris Impulse Response Function (DIRF) and leakage coefficient parameters. Convolution-based modeling which corrected for these signal contributions indicated that ^111^In activity was quantitative of cell viability with half-lives within 20 hrs to 37 days. We determine if the 37-day upper limit remains valid for endocardial injections by comparing previous epicardial cell leakage parameter estimates to those for endocardial cells. *Methods*. Normal canine myocardium was injected (^111^In-tropolone) epicardially (9 injections) or endocardially (10 injections). Continuous whole body and SPECT scans for 5 hours were acquired with three weekly follow-up imaging sessions up to 20–26 days. Time-activity curves evaluated each injection type. *Results*. The epicardial and endocardial kinetics were not significantly different (Epi: 1286 ± 253; Endo: 1567 ± 470 hours *P* = .62). *Conclusion*. The original epicardial estimate of leakage kinetics has been validated for use in endocardial injections.

## 1. Introduction

Apart from the long-term impact stem cells can have on the myocardium of the infarcted heart [1–3], progression in stem cell imaging requires an understanding of viability and function of the transplanted cells soon after transplantation. Some of the advancements in preclinical imaging technologies, namely, reporter gene imaging, to interrogate the status of transplanted cells [4, 5] cannot be currently applied clinically due to safety concerns related to injecting cells with foreign DNA. However, to this end, our group has reported on a SPECT cellular imaging technique using a clinically available radiotracer to determine the viability of transplanted cells as a function of time in a large animal model of acute myocardial infarction [6].

The basis of this technique is the radiotracer Indium-111 and its associated clearance kinetics in canine myocardium under different conditions. ^111^In (chelated with oxine, tropolone, etc. for in vitro cell labeling) is an FDA approved radiotracer [7] and is used in clinical infection imaging where white blood cells are radiolabeled ex vivo using the chelator, and reinjected cells localize to sites of infection and/or inflammation [8]. For the use of this tracer for imaging transplanted cells, in vivo acquired parameters called the leakage coefficient (C) and DIRF (Debris Impulse Response Function) are obtained to estimate the clearance half-lives of transplanted cells that can be measured from the SPECT signal. Using a convolution-based method, both parameters are used to derive the SPECT signal associated with viable cells only and constitute the upper (leakage coefficient) and lower (DIRF) bounds within which this method of estimating viable transplanted cell half-life is valid. While DIRF reflects the function by which ^111^In-labeled cytosolic proteins are cleared from the interstitial compartment of the myocardium (normal and infarcted) for example, cell death, an assumption in this method is that all interstitial ^111^In is removed according to DIRF regardless of the mechanism by which it enters this compartment. Similarly, C reflects the rate of ^111^In loss from radiolabeled cells into the interstitium as a result of cellular processes unrelated to cell death, which according to the aforementioned assumption, is then cleared from the myocardium according to DIRF. These upper and lower bounds then stipulate that if clearance half-lives of transplanted ^111^In-labeled cells are faster or slower than DIRF or C, respectively, the model cannot be applied to derive the transplanted cell half-life. 

Further in vitro work established the stability of ^111^In in the cell population of interest and the inability of ^111^In released from dead or viable cells to be resequestered by other cells modeling the surrounding myocardium. In vivo imaging with ^111^In established a correction curve whereby the measured SPECT signal from labeled cells transplanted into the myocardium could be used to estimate and remove ^111^In located outside viable cells generating a new biological half-life associated with viable cells. Finally, our group proposed a patient imaging protocol for clinical application of this technique [6]. 

 Given that cell number at targeted locations can correlate with therapeutic benefit [9, 10], direct intramyocardial injections of cells have shown higher relative retention [11] and is likely the preferred method for larger cells. For example, mesenchymal cells can plug capillaries when administered via the intracoronary route [12] resulting in microinfarction in canines [13]. Our cellular imaging method was tested using the stromal fraction of adult canine bone marrow administered through epicardial injection, which is surgically invasive and not likely to be used in routine clinical practice. As an alternative approach, however, endocardial catheter transplantation of cells would be a better solution for clinical cell transplantation and provides the option of multiple transplants over time. Importantly, differences in perfusion and intramyocardial pressures between the subepicardium and subendocardium in canine myocardium lead to transmural gradients [14, 15]. Retention of injected material may be influenced by the cyclical beat-to-beat changes in intramyocardial pressures with resultant loss of the injected material through needle tracks [16]. Reducing this significant initial loss will be instrumental in improving cell retention. Hence the forces that influence cell loss for an epicardial injection, in theory, would be different than those affecting an epicardial injection thus affecting clearance kinetics. 

The aim of this paper is to validate that the model leakage parameter for endocardial injections is similar to epicardial injections and comparatively evaluate the efficiency of the endocardial injection. This would allow wider application of the developed model as endocardial injections, from a clinical perspective, are much more likely to be used.

## 2. Materials and Methods

### 2.1. Intramyocardial Injections

Canine studies were approved by the Animal Use Subcommittee at the University of Western Ontario. A total of 19 separate injections of ^111^In-tropolone were administered into normal left ventricular myocardium of 12 healthy female canines (18–25 kg). Seven of 12 canines had 2 injections of ^111^In-tropolone with at least 2 weeks separating the last imaging session of the first injection and the first imaging session or the second injection which ensured that at least 10 half-lives had passed prior to the second injection. 

In the endocardial group, ^111^In-tropolone (mean ± SD: 50.8 ± 12.3 MBq; 10 injections) was injected into the anteroapical wall of the endocardium. In preparation for injection, canines were induced with propofol followed by endotracheal intubation and mechanical ventilation and anesthesia was maintained with isoflurane (1.5–2%). Endocardial injections were performed using the Stiletto Endomyocardial Injection System (Boston Scientific, Natick, MA) and the procedure is described elsewhere [17]. Using a 1 ml syringe containing ^111^In-tropolone (995 ul) and 0.5% India ink tissue dye to identify injection sites, 8–10 injections of ~0.1 ml each were delivered to the endocardium using the catheter (26-gauge needle). Each site of injection was recorded on transparencies. Following each injection, the needle and Stiletto catheter were retracted and advanced into another position, generally within 8–10 mm of the other injections. Within 30–40 minutes of the first injection, canines were transported to the SPECT suite. 

In the epicardial group, ^111^In-tropolone (52.1 ± 21.8 MBq; 9 injections; 25-gauge needle) was injected into the anteroapical wall. Following anesthesia as described above, a left thoracotomy was performed between the 3rd and 4th ribs exposing the surface of the heart. Several (6–10) intramyocardial injections were then performed with a syringe with a 25-gauge needle containing 1 ml of ^111^In-tropolone (including dye). Within 30 minutes of the injections, the thoracotomy incision was closed and the animal was transferred to the SPECT suite for imaging. Vital signs including heart rate were monitored throughout the injection and imaging sessions. At the final imaging session, animals were sacrificed using a bolus of KCl solution.

### 2.2. SPECT Acquisition and Analysis

On anesthetized canines, serial SPECT and wholebody images were acquired over the first 5 hours on injection day using a dual-head Millennium MG (General Electric, Milwaukee, WI) and a Symbia T6 SPECT/CT (Siemens, Erlangen, Germany) equipped with medium energy parallel hole collimators. Three subsequent SPECT follow-up imaging sessions were performed weekly over the following 3-4 weeks after the initial injection. Imaging parameters were as follows: 128 × 128 with 64 projections/head acquired over 180 degrees. Indium-111 counts were acquired with energy windows at 171 keV and 245 keV (±10%) for the GE system and 172 keV and 247 keV (±7.5%) for the Siemens system, and the acquisition time/projection angle increased from 30 seconds/projection on injection day to 30, 60, and 120 or 180 seconds/projection at 3 different followup time-points, respectively. Wholebody 2D images were alternately collected with SPECT on injection day using the same energy windows including one wholebody scan at each imaging follow-up. Wholebody imaging parameters: 256 × 1024 pixel matrix with 2.26 mm/pixel, fixed acquisition time of 23 minutes for the Millenium camera and 17 minutes for the Symbia (2.34 mm/pixel), and the same ^111^In energy windows as SPECT were used. 

Volume of interest (VOI) analysis was conducted on SPECT images that were corrected for background and subsequently reconstructed using an iterative algorithm [18]. The first SPECT data set acquired (*t* = 0) had a VOI defined as pixels ≥30% of the maximum pixel intensity and was used to create a mask image. This mask was then multiplied by each SPECT image acquired and the mean pixel intensity was determined (MATLAB, Mathworks, Natick, MA) [6]. Time activity curves (TAC) were generated for each injection and corrected for physical decay. Biexponential functions were fit to the TACs to determine the short (*T*_1/2_^*s*^) and long (*T*_1/2_^*l*^) components of ^111^In clearance from injected myocardium. Curve coefficients were also normalized to determine the fraction of the injected activity that cleared with the *T*_1/2_^*s*^ or *T*_1/2_^*l*^ and were reported as percentages. Heart to whole body (H : WB) activity ratios were also calculated from region of interest (ROI) analysis of wholebody scans using GE software (Xeleris, General Electric, Milwaukee, WI). Regions of similar area were drawn on wholebody images and counts derived from these regions were background corrected and normalized to the total image counts. These ratios were used for comparison between canines regarding the degree of radiolabel retention and are reported as a percentage. 

For the leakage parameter, *T*_1/2_^*l*^ data from our published report, which was similarly acquired, was compared to epicardial injections in this dataset and the combined epicardial data was compared to the endocardial data to determine if they were significantly different. 

### 2.3. Statistical Analysis

All statistical analysis was performed using SPSS 18.0 (SPSS Inc., Chicago, IL) with *α* set to 0.05. Non-parametric analysis was used to assess differences in the heart:wholebody ratios and *T*_1/2_^*s*^ and *T*_1/2_^*l*^ measurements using an independent samples test, and multiple pairwise comparisons were corrected using Bonferroni correction. All data are reported as the mean ± SEM.

## 3. Results

### 3.1. Evaluating Injection Efficiency in Canine Myocardium

**Figure 1:**
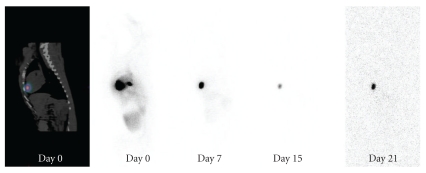
Images of ^111^In following epicardial delivery into normal myocardium following left thoracotomy. (Left) Sagittal SPECT/CT slice showing site of ^111^In injection in the myocardium. (Right) Wholebody ^111^In scans of canine demonstrating ^111^In activity at the injection site (day 0–15 wholebody scans are scaled to a maximum pixel value).

**Figure 2:**
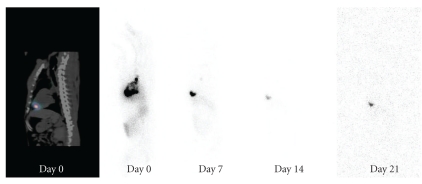
Images of ^111^In following endocardial delivery into normal myocardium with a specialized catheter under fluoroscopic guidance. (Left) Sagittal slice of SPECT/CT image localizing ^111^In injection within the myocardium on day of injection. (Right) Wholebody scans of same canine days 0, 7, 14, and 21 showing ^111^In within the myocardium (day 0–14 wholebody scans are scaled to a maximum pixel value).

The myocardial retention of ^111^In-tropolone following injection into canine myocardium was determined from serially acquired wholebody images, and ratios of heart to wholebody (H : WB) activity were calculated. Ratios (expressed as a percentage) were similar with the Epi group and Endo group having H:WB ratios of 48 ± 5% and 50 ± 4% (*P* = .902) respectively, directly following injection, indicating that the endocardial injection was as effective in tracer delivery as the epicardial injection within the specified time window of 30–40 min postinjection.  Table 1 shows the change in ratios for each animal for the first 5 hours following injection. Wholebody images from representative canines acquired after injection (Day 0 to 3 weeks) show the localization of ^111^In within the myocardial tissue as confirmed by SPECT/CT following epicardial (Figure 1) and endocardial (Figure 2) injection. Images also show the biodistribution of ^111^In within the liver, kidneys, and bladder. One animal in each of the groups had a lower ratio likely due to some injections into the left ventricular cavity rather than myocardium.

### 3.2. Early and Late Retention of Myocardial ^111^In: Viable Cell Leakage

Serial SPECT imaging on the day of injection followed by images acquired weekly over the subsequent 20–26 days helped to better define the early washout phase occurring immediately after the injections and the longer term retention of ^111^In within myocardial tissue. Biological half-lives generated from ROI analysis and fit with biexponential curves demonstrated *T*_1/2_^*s*^ and *T*_1/2_^*l*^ to be 2.17 ± 0.42 hours and 1567.07 ± 470.25 hours, respectively, for the endocardial group and 1.77 ± 0.25 hours and 1286.09 ± 253.02 hours for the epicardial group. Statistical analysis identified that there were no significant differences found when the *T*_1/2_^*s*^ (*P* = .594) and *T*_1/2_^*l*^ (*P* = 1.00) were compared between groups. The original, previously reported epicardial estimate of radiolabel leakage from viable cells was not significantly different from additional epicardial experiments (882.7 ± 242.8 (see [6]) hrs versus 1608.8 ± 369.5 hrs; *P* = .166).

**Figure 3:**
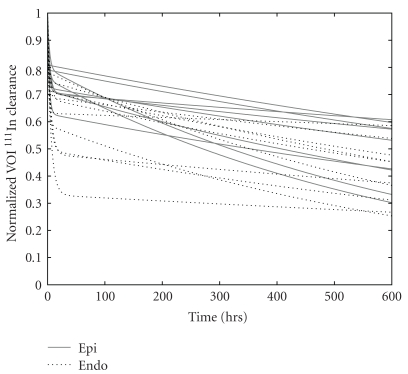
SPECT time-activity curves of ^111^In clearance from canine myocardium. Canines were directly injected with ^111^In-tropolone either by endocardial route via catheter (dotted lines) or epicardial route following thoracotomy (solid lines). Canines were serially imaged with SPECT on transplantation day with three follow-up imaging sessions. Region of interest analysis was conducted on images corrected for physical decay and background and fit to bi-exponential curves.

**Figure 4:**
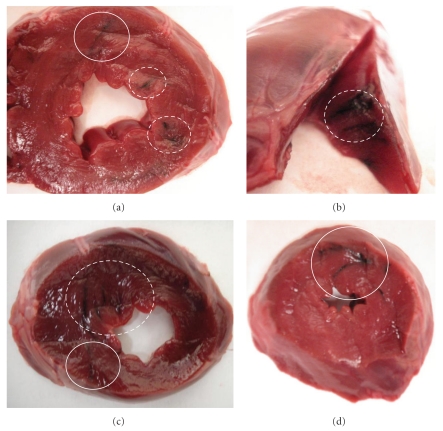
Digital images of ex vivo myocardial tissue confirming endocardial (dotted line circles) and epicardial (solid line circles) injections with tissue dye in normal tissue of representative canines. As noted during gross examination of the heart, needle punctures identified epicardial injection within the region of the left anterior descending coronary artery while endocardial injections were placed closer to the apex.

Curve fitting parameters are given in Table 2 and TACs are shown in Figure 3 for all canines. A significant difference was found between the long and short components for both endocardial group (*P* < .001) and epicardial group (*P* < .001). Of the epicardially injected activity that remained in the myocardium following the first SPECT acquisition approximately 40 minutes after the injection, on average 28% cleared the myocardium with the short half-life while the remaining 72% cleared with the longer half-life. Of the endocardial injections, 38% of the activity cleared the myocardium with the short half-life while 62% cleared with the long half-life thus indicating that the majority of the label injected was retained within the myocardial cells.  Figure 4 confirms SPECT images, and demonstrates myocardial tissue injection location as marked by tissue dye.

## 4. Discussion

Nonspecific imaging markers used for noninvasive cell tracking have their own perils. As outlined by Bengel et al. [19], clinical techniques typically used in understanding cell biodistribution over the short term prove difficulty over the longterm (i.e., weeks) as such markers can be susceptible to instability in the transplanted cell population or re-uptake of interstitial label into the surrounding tissue or even other transplanted cells making quantification more difficult. Label re-uptake has been demonstrated with iron oxide labels [20, 21] in stem cell transplantation studies, while other studies indicate SPECT radiolabels like ^99m^Tc-HMPAO [22] and PET tracers ^18^F-FDG [23] and ^64^Cu-PTSM [24] efflux rapidly from various populations of viable cells. 

In our previous work, we characterized the main sources of radiolabel loss from canine bone marrow stromal cells labeled with ^111^In-tropolone both in vitro and following injection through the epicardium. We identified transplanted cell death and radiolabel leakage from viable transplanted cells as main contributors and used their associated ^111^In clearance kinetics (*T*_1/2_^*l*^) for modeling the use of ^111^In as an in vivo marker of viability. For the determination of radiolabel leakage in vivo, we injected free ^111^In-tropolone in situ and monitored ^111^In clearance kinetics from endogenous normal myocardial cells as was done in the experiments reported here. Zhou et al. state that observed radioactivity cannot be translated to the number of surviving cells as ^111^In label released from dead cells may remain with the cell [25]. However, our model uses convolution as a means to correct for interstitial label related to death and leakage, and issues related to interstitial label are tenuous as we have verified that the clearance activity from dead cells is short relative to activity loss from viable cells with the average viable cell biological clearance ~79 times longer than that for dead cell clearance, or DIRF. To be clear, our model that incorporates these corrections for clearance of cellular debris and leakage from viable cells indicates that ^111^In can be used to quantitate cell half-life between 20 hrs and 60 days with the error in the measurements increasing as the upper and lower bounds are approached. In vitro work also indicated that dead cell activity or leaked activity is not taken up by either remaining viable transplanted cells or resident cardiomyocytes [6]. Additionally, our imaging protocol includes wholebody imaging to estimate the remaining absolute fraction of the injected dose in myocardium (H : WB ratio) and the observed *T*_1/2_^*l*^ estimates the biological half-life associated with viable transplanted cells. Numbers associated with the surviving fraction as a function of time can then be estimated providing the minimum cell number remaining, as our method cannot account for cells that proliferate subsequent to transplantation. Future work is needed to examine the robustness of this noninvasive approach to transplanted cell viability.

In this paper, we have further evaluated the use of the transendocardial injection technique using ^111^In-tropolone. Specifically, we have shown that the injection efficiency and retention characteristics of ^111^In-tropolone injected within normal canine myocardium are independent of the site of injection (i.e., subepicardium versus subendocardium). This is evident in the ^111^In clearance patterns reflected by *T*_1/2_^*s*^ and *T*_1/2_^*l*^. In the development of our technique for modeling ^111^In as an in vivo marker of transplanted cell viability, comparisons were necessary to demonstrate that differences in ^111^In leakage from myocardium were not injection-site dependent. Furthermore, we wanted to ensure that intrinsic differences between myocardial layers did not significantly affect *T*_1/2_^*s*^ which represents the mechanical loss associated with intramyocardial injection. Based on these results, it is expected that retention of endocardially injected cells may not be significantly different from those injected epicardially.

Transmural heterogeneity across the left ventricular heart wall has been extensively studied in the canine heart and may have implications for cell survival and retention. During ischemic injury, a well-observed phenomenon is the greater susceptibility of the subendocardium [26] which may be linked to higher oxygen demand within this myocardial layer [27] making it more vulnerable. Cell transplantation within peri-infarcted subendocardium with compromised flow may risk cell survival, and different flow patterns may themselves be a factor influencing cell clearance. Transmural intramyocardial pressure and perfusion gradients suggest higher contractility within the subendocardium with poorer perfusion [15] relative to the subepicardium in the canine [14, 27, 28] which could affect retention. Lower pressures in the LV cavity relative to the subendocardium [14] may also result in problems with retention of injected material allowing more material to leak into the cavity and redistribute cells to other organs. Optimization of transendocardial delivery suggest that parameters like needle length and injection volume can improve retention of various injectable materials [29, 30]. Irrespective of transmural differences, our data does not suggest differences in the leakage parameter between the subendocardial and subepicardial layers. Additional work with ^111^In labeled cells also support insignificant differences in retention and clearance kinetics in infarcted canine myocardium [17], but future work would also need to verify that similar clearance patterns exist for cellular debris between layers.

The radiotoxic effect of ^111^In for the purposes of tracking labeled cells has been previously addressed [31]. Reports indicate that the labeling mechanism of ^111^In is that it binds to cytosolic proteins having little direct contact with the nucleus [32]. In our study, large doses of ^111^In were injected into normal myocardium; however, radiotoxicity was not expected to be a primary concern considering the postmitotic nature of the adult canine myocardium. Previous work also evaluated the potential of fibrosis as a result of ^111^In radiotoxicity using delayed enhancement MRI following Gd-DTPA administration which was not positive [6].

In summary, we have confirmed the leakage component of our model demonstrating ^111^In retention in viable endocardial cells in vivo. The leakage gives the model an upper half-life limit of approximately 60 days. Efficiency of injections into the epicardium or endocardium is similar and physiological differences between myocardial layers do not factor into the kinetics of radiotracer clearance supporting the application of the model following endocardial transplantation.

## Figures and Tables

**Table 1:** Heart to wholebody ratios of ^111^In postinjection (ratios expressed as %).

EPI (%)										
Dog	1	2	3	4	5	6	7	8	9	
0 hrs	31.8	19.4	45.3*	49.2	62.6	56.4	59.7	57.8	49.5	
⋮	29.4	18.3	—	47.6	60.5	53.6	56.9		45.1	
⋮			—	46.8			55.0	54.1	42.9	
⋮	27.9	17.4	—	46.3	59.2	52.5	54.2			
⋮			—	46.2	57.2			51.4	41.0	
5 hrs	26.3	17.1	—	45.5	56.4	51.2	52.5	49.3	39.4	

ENDO (%)										
Dog	1	2	3	4	5	6	7	8	9	10

0 hrs	22.8	42.9	45.5	44.0	54.1	62.5	60.0	49.2	59.8	57.6
⋮	23.9	40.8	43.2	42.3	52.0	60.3	56.1	47.6	54.4	54.8
⋮	21.3	39.0	40.6	40.9	50.1	59.5	54.0	44.7	50.5	52.5
⋮	20.6	37.9	38.9		48.5	58.4	52.3	43.0	47.2	50.2
⋮	19.9	36.9	37.5	40.1	47.7	57.6	51.4	41.6	44.5	48.4
5 hrs	19.1	36.3	36.8	39.4	46.7	—	—	—	—	—

*Serial wholebody scans not acquired at day 0.

All data acquired over a 5 hr period following injection.

**Table 2:** Exponential curve fitting parameters.

*f*(*t*) = *a* · exp ^(−*bt*)^ + *c* · exp ^(−*dt*)^						
EPI						
Dog	*a* ^†^	*b*	*c* ^‡^	*d*	*T*_1/2_^*s*^ (hrs)	*T*_1/2_^*l*^ (hrs)
1	37.9	−0.3712	62.1	−0.0012930	1.87	537
2	16.0	−0.4705	84.0	−0.0017590	1.47	394
3	—	—	100	−0.0005308	—	1306
4	19.8	−1.004	80.2	−0.0005354	0.69	1295
5	29.2	−0.291	70.8	−0.0003566	2.38	1944
6	29.6	−0.2413	70.4	−0.0002413	2.87	2873
7	29.4	−0.4448	70.6	−0.0008787	1.56	789
8	28.2	−0.4850	71.8	−0.0005060	1.43	1370
9	36.6	−0.3662	63.4	−0.0006483	1.89	1069

			71.8 ± 2.6		1.77 ± 0.25	1286 ± 253

ENDO						

1	40.7	−0.3407	59.3	−0.0013530	2.03	512
2	31.5	−0.2468	68.5	−0.0006903	2.81	1004
3	21.8	−2.7770	78.2	−0.0012670	0.25	547
4	29.7	−0.3896	70.3	−0.0006643	1.78	1043
5	33.1	−0.4293	66.9	−0.0004125	1.61	1680
6	49.9	−0.2316	50.1	−0.0007734	2.99	896
7	25.0	−1.1510	75.0	−0.000840	0.60	825
8	37.6	−0.4884	62.4	−0.0001245	1.42	5567
9	63.0	−0.1564	37.0	−0.0003590	4.43	1931
10	51.3	−0.1858	48.7	−0.0004166	3.73	1664

			61.6 ± 4.1		2.17 ± 0.42	1567 ± 470

^†^Coefficients “*a*” and “*c*” normalized to 100%.

^‡^Dog 3 normalized data not included in fractional washout averages.
